# Oral Administration of *Spirulina platensis* at Early Gestation Modulates Litter Size and the Expression of Inhibin, Insulin, IGF-I, CO Q10, and BMP-15 in Ewes Induced for Twinning

**DOI:** 10.1155/2023/7682533

**Published:** 2023-01-12

**Authors:** Moustafa Zeitoun, Mohamed Ali, Tariq Almundarij, Emad Mousa, Ahmed Alghonaim

**Affiliations:** ^1^Department of Animal Production and Breeding, College of Agriculture and Veterinary Medicine, Qassim University, Buraydah, Saudi Arabia; ^2^Department of Animal and Fish Production, Faculty of Agriculture, Alexandria University, El-Shatby, Alexandria, Egypt; ^3^Department of Veterinary Medicine, College of Agriculture and Veterinary Medicine, Qassim University, Buraydah, Saudi Arabia; ^4^Ministry of Environment, Water and Agriculture, Riyadh, Saudi Arabia

## Abstract

Gestation in sheep necessitates the support of nutrients to avoid early embryonic mortalities. Therefore, this study investigates the effects of supplementing either L-arginine or Spirulina alga in the first trimester on the pregnancy rate, litter size, inhibin, insulin, IGF-I, CO Q10, and bone morphogenetic peptide 15 (BMP15) in maternal circulation. Animals were offered barley (500 g/head/day, 14% CP), alfalfa hay (1 kg/head/day, 12% CP), clean water, and balanced salt block licks as free choices. Forty *Noemi* and *Najdi* ewes were randomly allotted into three groups: control (C, *n* = 8), L-arginine (ARG, *n* = 16), and Spirulina (SP, *n* = 16). All females were implanted with CIDR for ten days. On days 8, 9, and 10, treated ewes were given a protocol comprised of human recombinant FSH at descending doses (50, 50, 40, 40, and 30, 30 IU, A.M, and P.M, respectively). At the fifth dose, animals were given an equivalent dose of hCG (240 IU). After CIDR withdrawal, ewes were exposed to fertile rams for mating. SP-ewes were orally given 50 ml (2%) Spirulina, and ARG-ewes were given 50 ml (35 mg/kg BW) L-arginine daily for 50 days postbreeding. Blood inhibin, insulin, IGF-I, CO Q10, and BMP15 were determined throughout gestation until parturition. The findings indicated that the conception rates were 25, 75, and 87.5% in C, ARG, and SP, respectively (*P* < 0.05). The percent of ewes giving birth to twins was 0, 25, and 50% in C, ARG, and SP, respectively (*P* < 0.05). The survival rates were 100, 81.8, and 83.3%, respectively. Birth weight was 5.6, 3.2, and 3.4 kg in C, ARG, and SP, respectively. Weaning weights were 28.3, 25.6, and 27.2 kg in C, ARG, and SP, respectively. BMP-15 was reduced (*P* < 0.05) in ARG than in C and SP. However, SP decreased (*P* < 0.05) inhibin more than in C and ARG. ARG and SP increased (*P* < 0.05) insulin than in C, whereas SP decreased (*P* < 0.05) IGF-I. SP increased CO Q10 compared with ARG. Ewes bearing twins revealed higher (*P* < 0.05) IGF-I (8.57 ng/ml) than those bearing singles (4.63 ng/ml); however, BMP-15 was higher in single (796.6 pg/ml) than in twin-bearing (387.5 pg/ml) ewes. In conclusion, providing early-gestating ewes with Spirulina enhances maternal health, productivity, and reproductive outcomes.

## 1. Introduction

Maximizing the productivity of the animal unit became a necessity in the current era of pandemics and political conflicts. This target drew researchers' attention to animal production due to the inflation of animal production supplies, particularly feedstuffs, vitamins, minerals, vaccines, and other items. Lack of a balanced ration for an animal causes a decrease in its productivity and reductions in its productive lifespan and reproductive performance [1]. Sheep flocks in the Arabian Gulf region constitute the primary source of meat since the inhabitants of this area consume mainly lamb and goat meat. L-arginine is an inducer for nitrous oxide production focused on various research aspects two decades ago [2]. Nutrients requirement during pregnancy have an indispensable role in neonatal health and the outcome of the ewe's production [3]. Several attempts have been conducted to maximize the productivity of farm animals using bioactive compounds like amino acids [2], probiotics [4], and microalgae [5]. The work of Fuller Bazer and his colleagues was the pioneer to highlight the necessity of L-arginine for early embryonic implantation, survival, and development in small ruminants [6–8]. Subsequently, Zeitoun and his associates orally administered L-arginine during the first 56 days postmating in *Najdi* ewes and obtained a heavier birth weight by 35% than control lambs [9]. This finding was later confirmed in mares, since L‐arginine supplementation in early pregnancy supported embryonic growth and the initiation of placentation [10].

Recently, research has focused on unconventional feed additives with valuable nutrients. These nutritious compounds include algae such as Spirulina and chlorella. *Spirulina platensis* was defined as filamentous cyanobacteria known as blue-green algae that act as single-cell proteins [11]. Spirulina contains (on a dry matter basis) a high percentage of protein (70%) and is rich in some amino acids like L-arginine (4.15%), glutamic acid (8.39%), lysine (3.03%), and methionine (1.15%), as well as vitamins such as A, B, E, and K [12]. Spirulina has been used as a diet additive in aquaculture [13, 14], chicken [15], dairy cows [16, 17], rabbits [18], and sheep [19]. Alghonaim and his group investigated the effects of Spirulina supplementation on the growth performance and digestibility parameters of sheep lambs [19].

Bone morphogenetic peptide-15 (BMP-15) is an oocyte-secreted peptide that modulates folliculogenesis, oocyte health, and ovulation outcome in humans [20]. This peptide was targeted to be related to fecundity in ewes [21]. Besides, CO Q10 was recently focused on as a bio-indicator for the healthy oocyte [22, 23]. CO Q10 was mentioned as a novel peptide that synthesized intercellular and played a vital role in the body as an electron transporter in the mitochondria [24]. The use of CO Q10 as a supplement for alleviating reactive oxygen species and improving the health of oocytes in women was confirmed recently [25].

To the best of our knowledge, no one has attempted to test the role of Spirulina supplementation in pregnant ewes during various stages of gestation. Our previous studies demonstrated that supplementing pregnant ewes at an early stage of pregnancy with L-arginine maintained the survival of the newborn lambs [26, 27]. Therefore, the objective of the present study was to explore whether supplementing a rich source of protein and antioxidants like Spirulina during early pregnancy in ewes would promote the implantation and subsequent survival of twin lambs, in addition to investigating the hormonal (i.e., inhibin and insulin) peptides (i.e., IGF-I and BMP-15) and antioxidant (CO Q10) expression in the ewes induced for twinning.

## 2. Materials and Methods

### 2.1. Animals and Location

Forty multiparous *Noemi* and *Najdi* ewes (average BW 45 ± 3.2 kg; age 26 ± 3.5 months) were housed in semi-shaded pens in the experimental station, College of Agriculture and Veterinary Medicine, Qassim University, in the Central region of KSA. Animals were offered barley (500 g/head/day), alfalfa hay (1 kg/head/day), clean water, and balanced salt block licks as free choices. According to the NRC requirements, this ration provides about 192 g crude protein (CP) per head per day [28]. Animals were submitted to the regular vaccination regime. The committee approved the animal handling and manipulation in this study of ethical animal care and welfare, Deanship of Scientific Research, Qassim University (contract#7804-1-1-2019Q), KSA.

### 2.2. Experimental Designs

Ewes were randomly allotted into three groups: control (C, *n* = 8) given the regular diet as mentioned above; ARG group ewes (*n* = 16) given the regular diet plus supplementation of L-arginine at a dose of 37.5 mg/kg BW/day for the 50 days following mating. SP ewes (*n* = 16) were given the regular diet plus 2.5 g/head/day *Spirulina platensis* powder dissolved in 50 ml distilled water for the first 50 days postmating.

### 2.3. Twinning Protocol, Natural Mating, and Parturition

All animals were exposed to intravaginal CIDR insertion for ten days; recombinant human FSH (GONAL-f, Serono, Bari, Italy) was administered (i.m.; AM and PM) in descending doses at 50, 50; 40, 40; and 30, 30 IU at days 8, 9, and 10 (a total dose of 240 IU, [26]). CIDR was removed on day 10 (AM; i.e., at the 5^th^ FSH dose), at which time an equivalent dose of hCG (240 IU, Pergnyl, Organon, Oss, Holland) was injected (i.m.). Ewes were subjected to fertile rams of the same breed for 72 hours. The dates and times of the onset of estrus and mating were recorded for each ewe. The estimated time elapsed between CIDR withdrawal and the onset of estrus was also recorded for each ewe. Ewes were examined for pregnancy by ultrasound machine (ALOKA SSD 500, Japan) with a 5 MHz endorectal linear array transducer 30 days postmating. The newborn's number, sex, survival rate, and weight were recorded at parturition.

### 2.4. Blood Sampling and Peptide and Hormone Determinations

Blood samples were collected via jugular venipuncture at days 0 (just before CIDR insertion), 8 (commencement of gonadotrophin administration), 10 (at CIDR withdrawal), at the onset of estrous and mating, days 7, 14, 21, and 28 postmating, and every other week thereafter until the day of delivery. Blood samples were collected in EDTA-Vacutainer® tubes, subjected to centrifugation (3000 rpm/3 min./5°C), and plasma was harvested and stored frozen (−20°C) until analyzed.

The study used commercial ELISA kits specific to sheep (My BioSource, CA, USA). Sheep insulin was determined by sandwich ELISA kit (Cat. No. MBS705810) with standard levels range 0–100 *μ* IU/mL, two *μ*IU/mL sensitivity, and intra- and interassay CV of 8.6 and 11.2%, respectively.

Sheep insulin-like growth factor-I (IGF-I) was determined in the samples by sandwich ELISA kits (Cat. No. MBS2122248). The assay used standard concentrations ranging from 0–60 ng/mL, a sensitivity less than 0.35 ng/mL, and intra- and interassay CV of 9.5 and 11.7%, respectively.

A competitive ELISA kit determined Sheep Bone Morphogenetic Protein 15 (BMP-15) (Cat. No. MBS736031). The standard concentrations range 0–5000 pg/mL, sensitivity 1.0 pg/mL, and intra- and interassay CVs are of 7.9 and 10%, respectively.

Sandwich ELISA kits were used to determine sheep inhibin (Cat. No. MBS743665). The standard concentration range was 0–2500 pg/mL with a sensitivity is of 1.0 pg/mL, and the intra- and interassay C.V were 6.9 and 9.8%, respectively.

Sheep Co Q10 was determined by a competitive ELISA kit (Cat. No. MBS7252409). The standard range was 0–100 ng/mL, the sensitivity was 1.0 ng/mL, and the intra- and interassay CV were 8.5 and 9.3%, respectively.

### 2.5. Statistical Analysis

Data of estrous, conception, and offspring number, birth, and weaning weights were analyzed by ANOVA. Data of the hormones and peptides were analyzed by least square analysis of variance for the repeated measures [29]. Mean comparisons were conducted through Duncan's multiple range test (DMRT). Differences were considered significant at *P* < 0.05. Data for discrete parameters were analyzed by the chi-square test according to SAS [29].

## 3. Results

Table 1 illustrates that ewes given FSH expressed higher percentages of estrus compared with C ewes. The time lapsed from CIDR removal until the onset of estrous was 56 ± 2.1, 36 ± 3.6, and 38 ± 4.3 hours (*P* = 0.03) in C, ARG, and SP, respectively. Conception rate was higher (*P* = 0.04) in SP and ARG than control. The number (%) of ewes that gave birth to singles was 2 (25%), 8 (50%), and 6 (37.5%) (*P* = 0.04) in C, ARG, and SP, respectively. However, the number (%) of ewes that gave birth to twins was higher (*P* = 0.03) in SP than in ARG and C. The failure of conception rate was higher in C than in ARG and SP (*P* = 0.03). The ratio of gender (male: female) among treatments was 100% males, (10M : 6F) and (12M : 10F) in C, ARG, and SP, respectively. No significant effect was found due to the sex of the offspring. The mean lamb birth weight was higher (*P* = 0.03) in C than in ARG and SP. In C, ARG, and SP, the percent survival at birth was 100, 81.25, and 81.8%, respectively. However, survival at weaning was 100% among treatments. The mean 90-day lamb weaning weight did not differ (*P* = 0.12) among treatments. Therefore, total kilograms of weaned lambs were higher in SP and ARG than in C. The mean number of weaned lambs per each treated ewe was higher in SP and ARG than in C.

**Figure 1:**
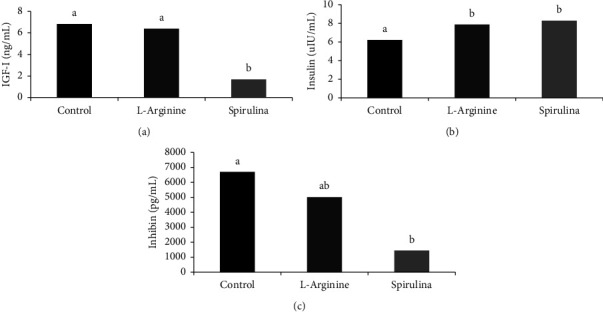
Effect of oral administration of Spirulina or L-arginine to sheep ewes induced for twinning on plasma IGF-I (a), insulin (b), and inhibin (c). (^a,b^*P* < 0.05).

As shown in Figure 1(a), Spirulina (1.69 ± 0.22 ng/mL) decreased (*P*=0.0001) IGF-I compared to the control (6.82 ± 0.75 ng/mL); however, arginine (6.39 ± 0.66 ng/mL) maintained IGF-I similar to the control. On the contrary, Figure 1(b) illustrates the level of insulin and reveals that L-arginine (7.88 ± 0.39 *μ*IU) and Spirulina (8.28 ± 0.19 *μ* IU) increased (*P*=0.03) insulin compared to control (6.22 ± 0.46 *µ*IU).

Figure 1(c). Illustrates that Spirulina significantly (*P* < 0.05) decreased (1441.57 ± 203.19 pg/mL) inhibin compared to control (6697.2 ± 977.12 pg/mL). However, L-arginine treatment did not differ in inhibin level (5012.55 ± 1255.02 pg/mL) than the control.

**Figure 2:**
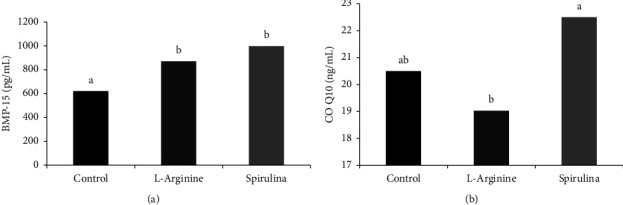
Effect of oral administration of Spirulina or L-arginine to sheep ewes induced for twinning on plasma BMP-15 (a) and CO Q10 (b). (^a,b^*P* < 0.05).

As shown in Figure 2(a), sheep BMP-15 level was lower (*P*=0.0001) in the ewes given L-arginine (621.22 ± 43.67 pg/mL), whereas Spirulina maintained BMP-15 (997.85 ± 37.61 pg/mL) comparable to the control (870.09 ± 99.14 pg/mL).

Figure 2(b) exhibits differences in the levels of CO Q10, in which Spirulina did not change the CO Q10 level (22.5 ± 0.5 ng/mL) than in the control (20.5 ± 1.43); however, L-arginine significantly (*P*=0.0033) decreased the CO Q10 level (19.03 ± 0.67 ng/mL).

The only significant correlation between the hormones and peptides was the positive correlation between IGF-I and inhibin (*r* = 0.71, *P* < 0.0001). However, there were non-significant correlations among other metabolites.

Ewes that gave birth to twins contained slightly higher (*P* = 0.08) levels of BMP-15 (963.8 ± 47.7 pg/mL) compared to ewes that gave birth to singles (796.6 ± 42.3 pg/mL), and both groups expressed significantly (*P* < 0.05) higher levels of BMP-15 than in ewes that did not give birth. Moreover, IGF-I levels were higher (*P* < 0.05) in the twin-(8.6 ± 0.9 ng/mL) than single- (4.6 ± 0.9 ng/mL) bearing or nonpregnant (3.3 ± 0.4 ng/mL) ewes. Contrariwise, CO Q10 concentrations were lower (*P* < 0.05) in twin- (16.8 ± 0.6 ng/mL) compared to single- (21.4 ± 0.6 ng/mL) or nonpregnant (21.5 ± 0.8 ng/mL) ewes. Inhibin and insulin concentrations did not alter (*P* > 0.05) due to the number of offspring.

## 4. Discussion

Inappropriate placental vascular development leads to insufficiency of nutrients and gas exchange to the fetus (es) from the mother, resulting in intrauterine growth restriction (IUGR) or early embryonic death [30]. L-arginine was proven to stimulate nitrous oxide (NO) production, which enlarges the placental blood vessels and promotes angiogenesis. This function results in excess nutrients and oxygen provision to the fetuses in their first trimester [31]. Administration of L-arginine at early gestation has previously been given to ewes bearing singles or twins and exhibited better implantation, fetal growth, development, and survival [26]. Whether the embryos at their early implantation and growth would require the amino acid L-Arginine per se or need a source of protein (i.e., containing L-arginine and other essential amino acids) for the synthesis of the enzymes and growth factors for a better uterine milieu and proper implantation was the focus of the current exploration.

*Spirulina platensis* has been scrutinized as a food and feed source for humans and animals. The main nutritious ingredients in Spirulina are; protein (∼60–70%) which is rich in sulfur-containing amino acids like methionine and cysteine; fats which possess a high percentage of polyunsaturated fatty acids (PUFA), like linoleic acid and *γ* linolenic acid (GLA); and minerals like potassium, phosphorus, calcium, ferrous, copper, and zinc [32]. Also, Spirulina was shown to be a toxin-free microalga. It has been reported that supplementing diabetic mice with Spirulina improved the percentage of fetal implantation [33]. Moreover, El-Ratel and Gaber obtained a larger litter size (60% higher than control) from Spirulina-supplemented diet to rabbit does (300 mg/kg diet) [34]. Moreover, Tilapia and Zebra fish provided with Spirulina increased egg production, vitellogenesis, hatching, and survival rates [35, 36]. A few recent attempts have been made to test the effects of supplementation with Spirulina on the growth and immunity of dairy suckling calves [37] and sheep lambs [19]. Remote data, so far, have been published on the effects of microalgae (i.e., Spirulina) on the reproductive efficiency of ruminants. The current findings confirm the surpass of Spirulina over L-arginine on the total lamb outcome, leading to the concept that this alga contains high levels of nutrients that meet the pregnant ewes' requirements during their early gestation. Provision of the Spirulina in the current study resulted in approximately 4.3 times lambs' crops at weaning per treated ewe as obtained from control ewes and ∼1.5 folds higher than that given by L-arginine. The antioxidant, immune-stimulants, and growth properties of Spirulina alga must not be overlooked [19, 37–39]. The high level of CO Q10 found in the SP-ewes in the current study would support the survival and higher implantation, postpartum growth rate, and high weaning weight. It has been previously stated that in hot climates, supplementation of rabbit diets at 300 mg Spirulina/kg diet improved the fetal developmental competence (*in vivo* and *in vitro*) and increased the litter size [34]. The anabolic potency of Spirulina was later revealed in growing lambs raised under heat stress and provided with an 8 mg Spirulina/kg diet for 90 days [19]. Meanwhile, another biological explanation for adding Spirulina was suggested by a Korean research team, which monitored the effect of nanoparticles of *Spirulina maxima* on the *in vitro* development of porcine oocytes. They concluded that adding SP nanoparticles to the oocytes in the *in vitro* maturation media increased the preimplantation porcine and cloned embryo development by increasing glutathione [40]. From another point of view, providing the mother with a rich source of energy pre- and during early pregnancy would augment fertilization, early cleavage, and early embryonic implantation and development [41]. Previous studies supported the concept that providing pregnant ewes [42] or young, growing lambs [38] with Spirulina enhanced offspring birth weight and lamb growth rate, respectively. Ewes given Spirulina in the current study revealed higher (*P* < 0.05) levels of insulin, CO Q10, and BMP15; however, the same ewes revealed lower levels of IFG-I and inhibin than control ewes. There found a strong positive correlation between inhibin and IGF-I, the relationship that explains the lower inhibin in peripheral blood is accompanied by lower IGF-I, which leads to higher healthy ovulated oocytes [43]. In control pregnant ewes, there was a decrease in IGF-I in the last 30–45 days near delivery; that means this growth factor might have played its role in fetal muscle development during early and mid-gestation [44–46]. Conversely, the current data revealed that Spirulina and L-arginine elevated maternal insulin levels above those found in control ewes. During pregnancy, the demand for insulin increases due to the interference between pregnancy hormones and insulin sensitivity, leading to a reduction in insulin sensitivity [47]. The increased insulin in the circulation of the ARG and SP ewes in the current study could facilitate fetal metabolism and provide a source of energy for the twins, ensuring their ongoing growth, development, and survival. Furthermore, Lunesu and his colleagues demonstrated that ewes exposed to diets differing in the quantity and quality of carbohydrates evoke insulin sensitivity during intrauterine and postnatal lives [48]. It has been confirmed that BMP-15 and GDF9 are major determinants of ovulation rate and litter size (i e., fecundity), and that these genes exist in prolific sheep breeds. The current concern is whether or not these growth factors are inherent to the prolific sheep or might exist in the ewes induced for twin/multiple births. The trend of high levels of BMP-15 in SP and LG ewes accounted for 1.6 and 1.4, more than control, respectively. This finding confirms the hypothesis of the relationship between BMP-15 and ovulation rate in spontaneous or induced ovulation. In this respect, Davis pointed out the existence of four mutants of the BMP-15 genes [21]. The current finding that the high concentration of BMP-15 coincided with the higher ovulation rate in SP ewes was previously stated by Davis and his associates [49]. They found that crossing Booroola Merino with Inverdale resulted in daughters having active ovaries and high ovulation rates, averaging 4.4 oocytes [49]. The two mutations, BMP-15 and BMPR-1B, increased ovulation by 44% and 90%, respectively. A recent study suggested a possible interaction between BMP15 and FecB/GDF on increasing ovulation rates in Luzhong mutton sheep in China [50]. CO Q10 has been shown to possess three main functions on the cellular level, namely, (i) transfer of reducing equivalents in the electron transport chain, (ii) generation of superoxide anion radical, O2^*∗*^−, and (iii) quenching of free radicals [51]. It has been proved that supplementation of CO Q10 revealed a potent antioxidant function leading to reduced free radicals in the exercised subjects [52]. In a study on mice, supplementing females suffering from preeclampsia with CO Q10 alleviated the adverse symptoms, improved health, and increased the number of pups born surviving [53]. The capability of CO Q10 as an antioxidant for improving human oocyte quality via its beneficial impact on the follicular level has been confirmed [54]. The current finding exhibited the excellence of Spirulina over L-arginine in elevating CO Q10 in maternal circulation. This observation would augment the excess growth of fetuses in SP compared to ARG-treated ewes.

In conclusion, providing pregnant ewes during their early stage of gestation with 2.5 g *Spirulina platensis* per a head per day for the first 50 days postbreeding would enhance the twin-bearing maternal health, fertilization, implantation, and pre- and postnatal survival of fetuses in estrous-synchronized/induced twinning *Najdi* and *Noemi* ewes. Also, supplementing pregnant ewes with the said dose of Spirulina kept the hormones, fecundity peptide, and the antioxidant CO Q10 at levels sufficient to maintain the offspring's survival and postnatal growth. Further studies using a larger sample size of ewes and testing the provision of Spirulina alga and its micronutrients during the three trimesters of gestation are warranted.

## Figures and Tables

**Table 1:** Effect of supplementation of L-Arginine and Spirulina on the reproductive traits of *Najdi* and *Noemi* ewes induced for twinning.

Trait	Control	Arginine	Spirulina
No. ewes within the treatment	8	16	16
Estrous exhibition (%)	80	100	100
Time from CIDR removal until the onset of estrus (h)	56 ± 2.1	36 ± 3.6	38 ± 4.3
Conception rate (%)	25	75	87.5
No. stillborn lambs	0	3	4
Birth weight (kg)	5.6 ± 0.2	3.2 ± 0.3	3.4 ± 0.15
No. (%) ewes gave no birth	6 (75%)	4 (25%)	2 (12.5%)
No. (%) ewes delivered singles	2 (25%)	8 (50%)	6 (37.5%)
No. (%) ewes delivered twins	0 (0%)	4 (25%)	8 (50%)
No. live lambs/treatment	2	13	18
Mean no. weaned lambs/treated ewe	0.25	0.813	1.125
Survival at weaning (%)	100	100	100
Weaning weight (kg)	28.3 ± 0.3	25.6 ± 0.2	27.2 ± 0.2
Total weaned lamb weight/treatment (kg)	56.6	332.8	489.6
Mean kilograms of lamb at weaning/treated ewe	7.08	20.8	30.6

## Data Availability

The data used to support the findings of this study are available from the corresponding author, upon reasonable request.
